# Supplementary material for: Interfacial Compatibilization into PLA/Mg Composites for Improved In Vitro Bioactivity and Stem Cell Adhesion
Source: Molecules. 2021 Sep 30;26(19):5944. doi: 10.3390/molecules26195944 (PMC8512483; doi:10.3390/molecules26195944)
Supplement: Supplementary file 1 [file molecules-26-05944-s001.zip › molecules-1371668-supplementary.pdf]

molecules

MDPI

Supplementary Materials

Interfacial Compatibilization into PLA/Mg Composites for Improved In Vitro Bioactivity and Stem Cell Adhesion

Meriam Ben Abdeljawad [1], Xavier Carette [1], Chiara Argentati [2], Sabata Martino [2], Maurice-François Gonon [3], Jérémy Odent [1], Francesco Morena [2], Rosica Mincheva [1,*] and Jean-Marie Raquez [1,*]

[1] Laboratory of Polymeric and Composite Materials (LPCM), Center of Innovation and Research in Materials and Polymers (CIRMAP), University of Mons, 23 Place du Parc, 7000 Mons, Belgium; Meriam.benabdeljawad@umons.ac.be (M.B.A.); xa92@hotmail.com (X.C.); jeremy.odent@umons.ac.be (J.O.)
[2] Department of Chemistry, Biology and Biotechnology, University of Perugia, 06123 Perugia, Italy; chiara.argentati@unipg.it (C.A.); sabata.martino@unipg.it (S.M.); francesco.morena@unipg.it (F.M.)
[3] Department of Material Sciences, University of Mons, 20 Place du Parc, 7000 Mons, Belgium; mauricefrancois.gonon@umons.ac.be
* Correspondence: rosica.mincheva@umons.ac.be (R.M.); jean-marie.raquez@umons.ac.be (J.-M.R.); Tel.: +32653734-57 (R.M.); +3265373480 (J.-M.R.)

Table S1. DMTA results of PLA/xMg and PLA/10Copo/xMg composites (x = 0, 5, 10 and 15 wt. %) at 37 °C.

	**PLA/xMg**			**PLA/10Copo/xMg**		
**Mg amount (wt.%)**	**E′(GPa)**	**$T_{Tan\delta}$ (°C)**	**Tanδ**	**E′(GPa)**	**$T_{Tan\delta}$ (°C)**	**Tanδ**
X=0	2.0	59	2.52	1.5	57	2.28
X=5	2.1	62	2.62	1.7	53	2.12
X=10	2.5	61	2.60	2.1	54	2.11
X=15	2.3	58	2.55	1.6	50	2.17

Table S2. Thermal properties of PLA and the different composites.

Samples	T_g (°C)	T_c (°C)	T_{m1} (°C)	T_{m2} (°C)
PLA	62	117	165	171
PLA/5Mg	61	116	164	170
PLA/10Mg	62	117	164	170
PLA/15Mg	62	116	163	169
PLA/10Copo	53	93	-	169
PLA/10Copo/5Mg	53	92	160	168
PLA/10Copo/10Mg	55	91	159	167
PLA/10Copo/15Mg	54	90	156	165

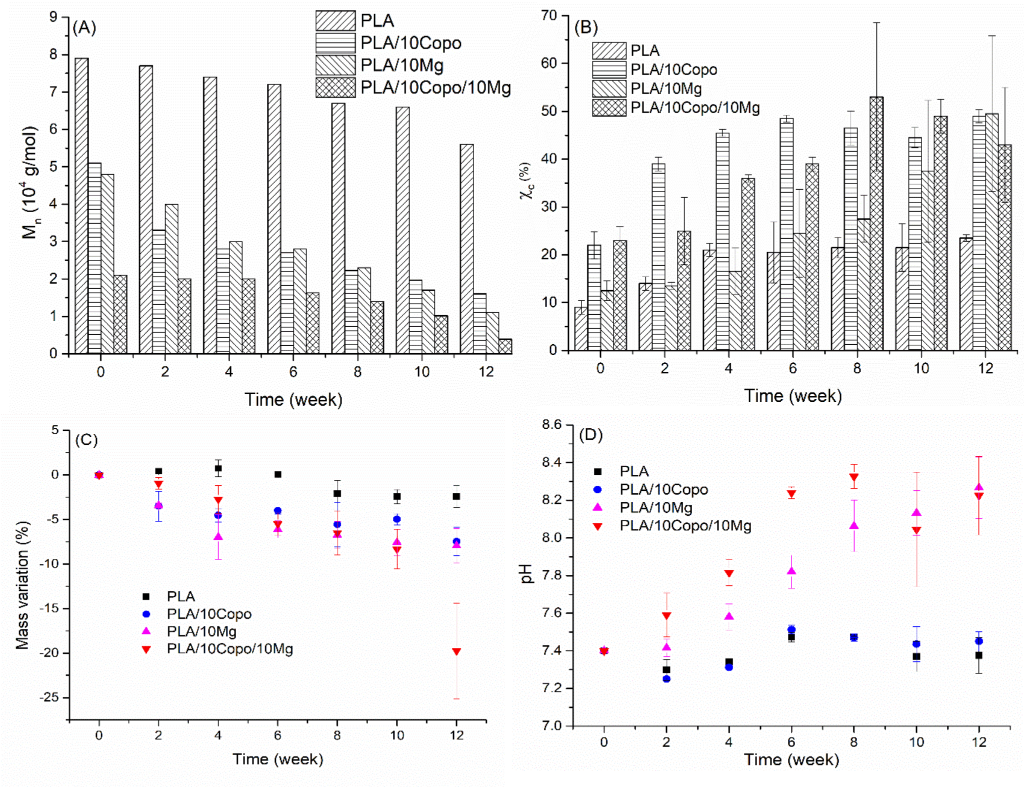

Figure S1. Evaluation of (A) GPC, (B) crystallinity (first heating, 10°C/min), (C) Mass and (D) pH of different samples during the degradation in SBF during 12 weeks.

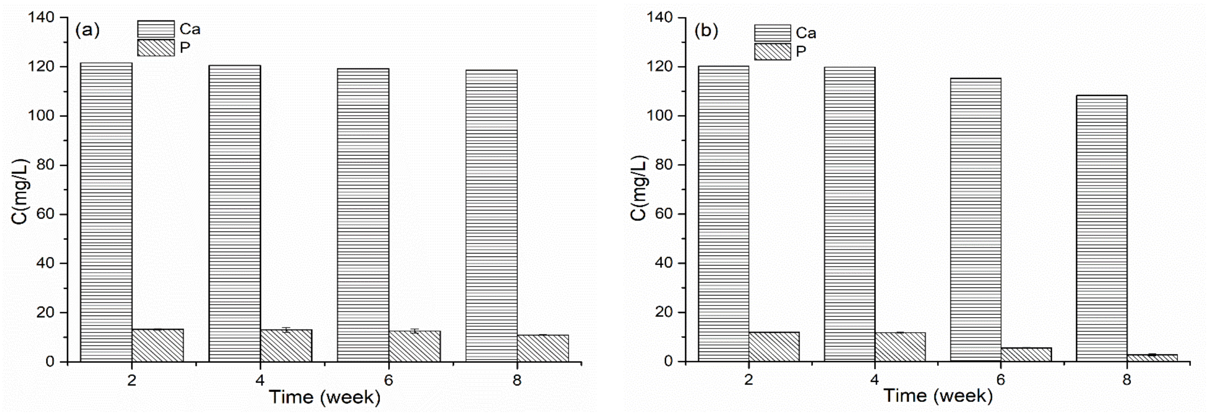

Figure S2. Ions concentration changes (Ca, P) during the degradation process in SBF (a) PLA/10Mg and (b) PLA/10Copo/10Mg composites. Error bars in (a) PLA/10Mg: Ca [0.288 - 0.969] mg/L; P [0.040 - 0.058] mg/L and in (b) PLA/10Copo/10Mg: Ca [0.172 - 0.473] mg/L; P [0.005 - 0.038] mg/L.

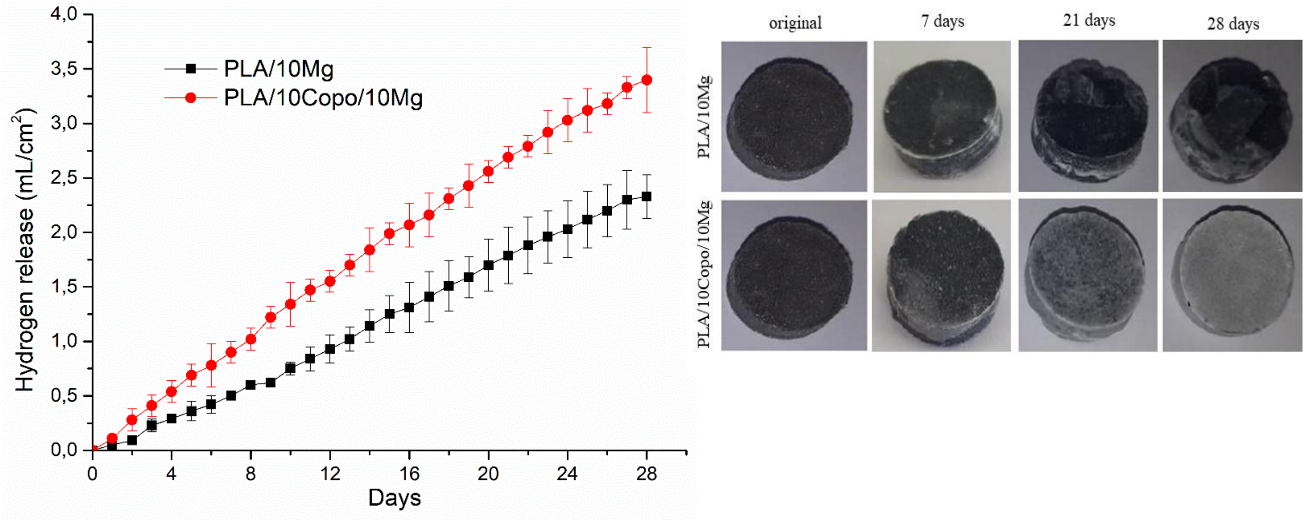

Figure S3. (left) Accumulation amount of hydrogen release as a function of immersion time in PBS and (right) the visual aspect of the composites.

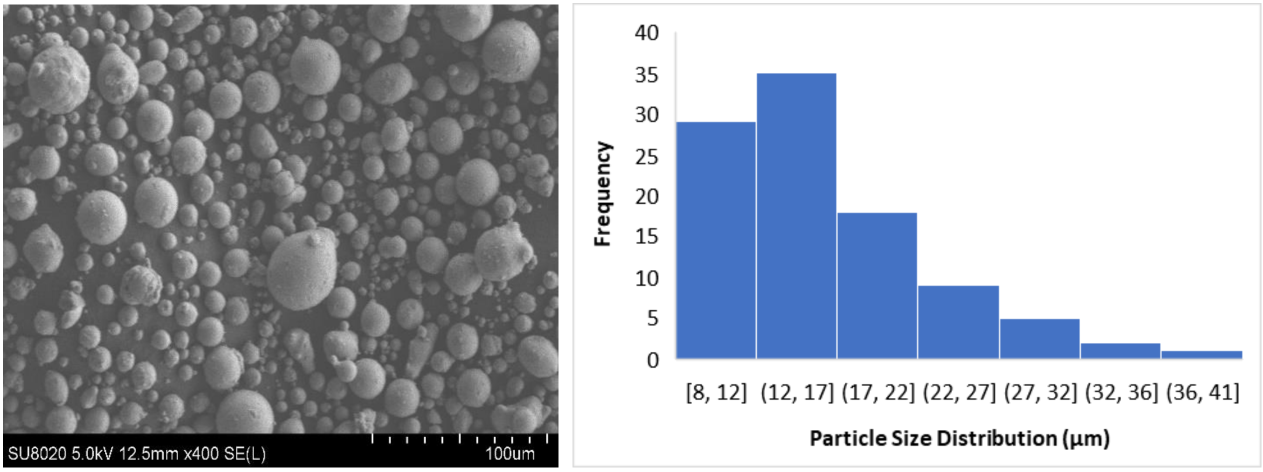

Figure S4. (left) SEM image and (right) size distribution of Mg microparticles.

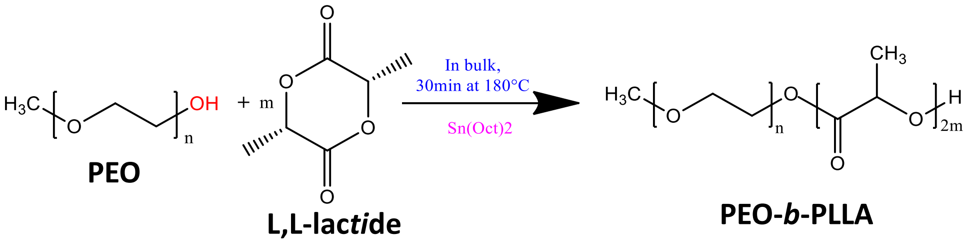

Figure S5. Synthesis of PEO-*b*-PLLA diblock copolymer by ROP of L,L-lactide.

Table S3. Nominal Ion Concentrations of the SBF (pH=7.4) in Comparison with that of human blood plasma (pH=7.2-7.4) (mM) ion.

**Ion**	**Na^{+}**	**K^{+}**	**Mg^{2+}**	**Ca^{2+}**	**Cl^{-}**	**HCO_3^{-}**	**HPO_4^{2-}**	**SO_4^{2-}**
Plasma	142.0	5.0	1.5	2.5	103.0	27.0	1.0	0.5
SBF	142.0	5.0	1.5	2.5	147.8	4.2	1.0	0.5

A

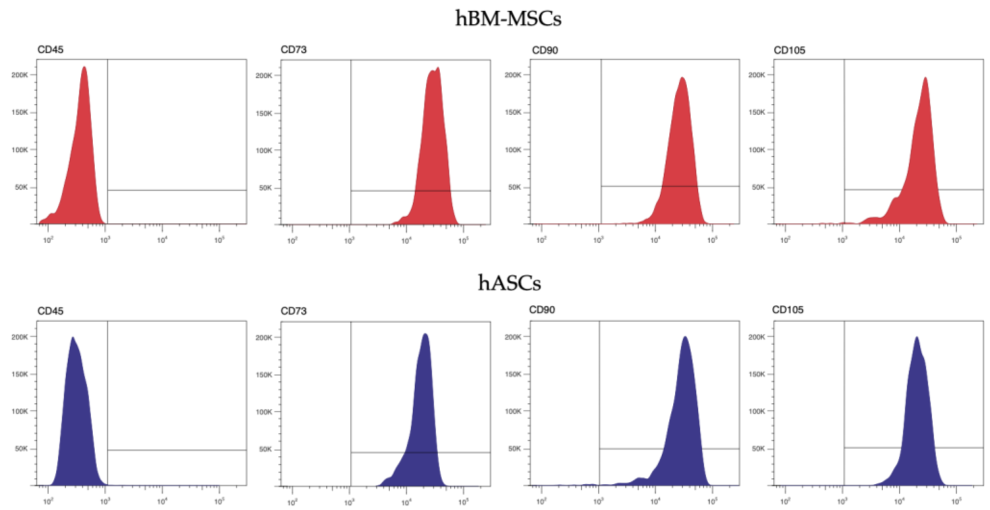

B

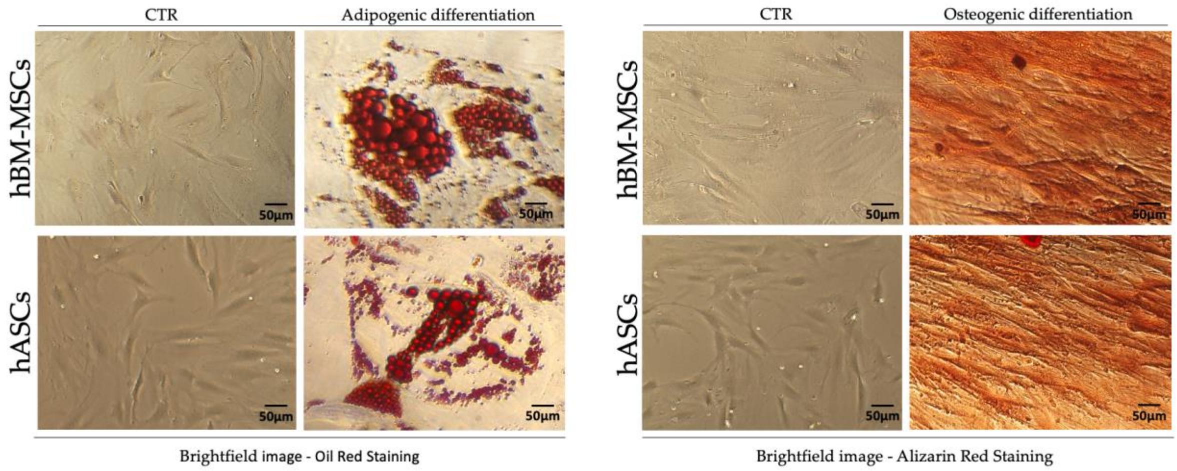

Figure S6. (A) Phenotypical characterization of MSCs showing the negativity of CD45 expression and the positivity of CD73, CD90, and CD105. (B) Multipotential properties of hBM-MSCs and hASCs after adipogenic (left) and osteogenic induction (right).
